# MST1/2 in inflammation and immunity

**DOI:** 10.1080/19336918.2023.2276616

**Published:** 2023-11-01

**Authors:** Tongfen Li, Yiqiong Wen, Qiongfen Lu, Shu Hua, Yunjiao Hou, Xiaohua Du, Yuanyuan Zheng, Shibo Sun

**Affiliations:** Department of Pulmonary and Critical Care Medicine, First Affiliated Hospital, Kunming Medical University, Kunming, China

**Keywords:** Immunity, inflammation, the mammalian Sterile 20-like 1, the mammalian Sterile 20-like 2, pathways

## Abstract

The mammalian Sterile 20-like kinase 1/2 (MST1/2) belongs to the serine/threonine (GC) protein kinase superfamily. Collective studies confirm the vital role MST1/2 in inflammation and immunity. MST1/2 is closely related to the progress of inflammation. Generally, MST1/2 aggravates the inflammatory injury through MST1-JNK, MST1-mROS, MST1-Foxo3, and NF-κB pathways, as well as several regulatory factors such as tumor necrosis factor-α (TNF-α), mitochondrial extension factor 1 (MIEF1), and lipopolysaccharide (LPS). Moreover, MST1/2 is also involved in the regulation of immunity to balance immune activation and tolerance by regulating MST1/2-Rac, MST1-Akt1/c-myc, MST1-Foxos, MST1-STAT, Btk pathways, and lymphocyte function-related antigen 1 (LFA-1), which subsequently prevents immunodeficiency syndrome and autoimmune diseases. This article reviews the effects of MST1/2 on inflammation and immunity.

## Introduction

The mammalian Sterile 20-like (MST) kinases belong to the serine/threonine (GC) protein kinase superfamily [1]. MST kinases are evolutionarily conserved homologue of yeast Sterile 20 (STE20) kinase 1 [2–4]. In mammals, there are five kinds of MST kinases, including MST1 (also known as STK4), MST2 (STK3), MST3 (STK24), MST4 (STK26), and YSKI (STK25 or SOK1) [1]. In addition, MST kinases play an important role in the mammalian cells proliferation, migration and apoptosis, immune regulation, and inflammatory response [1,2].

As a class II GC protein kinase [5], MST1/2 is the main component of the mammalian Hippo signaling pathway [2] which regulates the proliferation and survival of cells [6–9]. Increasing studies suggest that MST1/2 is widely involved in the development or prognosis of diseases that relate to inflammatory [10–12] and immune [13,14]. Accordingly, the effects of MST1/2 on inflammation and immunity are reviewed in this paper.

## Structure and function of MST1/2

### The structure of MST1/2

MST1 is composed of 487 amino acids [9] and contains the N-terminal catalytic domain of class STE20 which is followed by a non-catalytic tail containing, successively, an autoinhibitory segment, and a coiled-coil domain [15] mediating dimerization [16]. MST2 is composed of 491 amino acids which is the accessory nucleotide of MST1 [16]. Accordingly, MST2 is closely related to MST1.

There is a unique helical Salvador-Rassf-Hippo (SARAH) domain near the carboxyl terminal of MST1/2 [5]. In the non-phosphorylated state, SARAH domain stably interacts with other peptide substrates which contain SARAH domain for homodimerization and heterodimerization [5] to regulate the signal transduction of MST1/2 [17].

### The function of MST1/2

#### The role of MST1/2 in inflammatory response

It is reported that current researches on MST1/2 involving in the inflammatory response are mainly focused on myocardial cell injury [18]. Overexpression of MST1 promotes excessive inflammatory reaction of the myocardial cells [19] to promote the myocardial cell necrosis [18,20], which induces myocardial fibrosis and cardiac hypertrophy [21]. MST1-mROS signaling pathway is activated by suppressor of ras val-2 (SRV2) [22] which is up-regulated after hypoxia treatment [18], thus aggravating the inflammation of myocardial cells [18] to promote the death of the myocardial cells [23]. MST1 catalyzes the myocardial inflammation in diabetic mice, the activation of MST1 is partially inhibited by chronic exercise training to relieve response of inflammatory [24]. In addition, with MST1 is deficient, the levels of inflammatory factors decreases in the myocardial cells treated by hypoxia in vitro, mitochondrial homeostasis is maintained, mitochondrial fission is inhibited, thus inhibiting the inflammatory reaction of the myocardial cells, and the survival of the myocardial cells being promoted [19]. The reperfusion therapy of XMU-MP-1 inhibits the phosphorylation of MST1 [25], protecting the myocardial cells [12,24,25].

Furthermore, MST1 aggravates the secondary brain injury (SBI) following the intracerebral hemorrhage (ICH) [12]. XMU-MP-1 reperfusion therapy effectively inhibits the activation of downstream protein P-LATS1 and P-YAP of MST1 to reduce the neuronal cell death and the inflammatory reaction in rats with ICH to alleviate SBI [12].

MST1 aggravates the poor prognosis of spinal cord injury [26,27]. On the one hand, MST1 damages the mitochondrial function by attenuating the phosphorylation of adenosine phosphorylation-activated protein kinase α (AMPKα) to promote NF-κB-related inflammatory reaction in the injured spinal cord [26]. On the other hand, MST1 damages the mitochondrial function to reduce the formation of ATP by promoting the release of cytochrome c and the activation of caspase-3, which strengthens the mitochondrial-mediated apoptosis pathway and promotes the activation of microglia and glial cells to enhance the local inflammatory response after the spinal cord injury [27]. The miR-139-5p inhibits the expression of MST1 and accelerates the functional recovery of the injured spinal cord to promote the survival of neurons [26].

**Figure 1.**
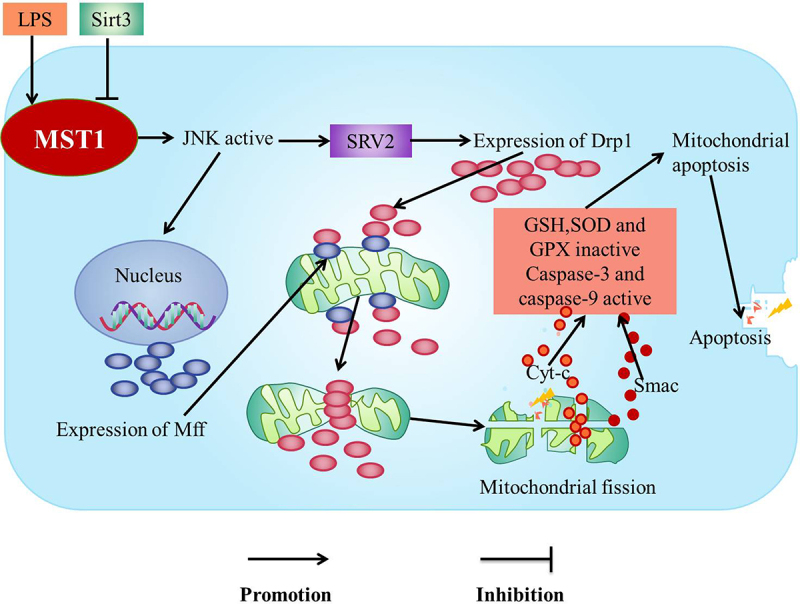
MST1-JNK pathway.

The lipopolysaccharide (LPS)-mediated MST1-JNK signaling pathway increases the levels of SRV2 which induces mitochondrial fission to enhance the inflammatory response of mouse microglia cells (BV-2) and inhibition of MST1-JNK signaling pathway by Sirtuin 3 (Sirt3) to protect BV-2 microglia from inflammation-mediated cell damage [22] (Figure 1).

JNK c-Jun N-terminal kinase; LPS Lipopolysaccharide; SRV2 Suppressor of ras val-2; Drp1 Fission-promoting factor mitochondrial Kinetic associated protein; Mff The drp1 receptor; Cyt-c, Smac The mitochondrial pro-apoptotic mediators; Caspase-3, caspase-9 The apoptotic executive; GSH Glutathione; SOD Superoxide dismutase; GPX Glutathione peroxidase; Sirt3 Sirtuin3; mPTP mitochondrial permeability transition pore

LPS up-regulates the activity of MST1 to promote the activation of JNK pathway, leading to the up-regulation of SRV2, which subsequently increases the level of Drp1. Furthermore, the activated JNK is transported to the nucleus to increase the expression of Mff. Drp1 migrates from the cytoplasm to the mitochondrial surface and binds with Mff on the mitochondrial outer membrane to recruit Drp1. Drp1 subsequently forms a GPT-dependent contraction ring which divides the mitochondria into several fragments to promote the mitochondrial fission, the mitochondrial potential collapse and mPTPs opening. Cyt-c and Smac leak into the cytoplasm after mPTPs are opened to active the apoptotic executive caspase-3, caspase-9 and rapidly down-regulate the activities of SOD, GSH, and GPX, which lead to the mitochondrial apoptosis, thus the apoptosis of the myocardial cells and BV-2 cells occur.

In addition, MST1 promotes tumor necrosis factor-α (TNF-α) inducing the injury of the nasal epithelium through increasing the inflammation [28]. The deletion of MST1 alleviates the inflammatory injury of the nasal epithelium [29].

*MST1/2 inhibits inflammatory injury*. However, the role of current MST1 in the inflammation remains contradictory. Some authors have suggested that MST1/2 may be a factor in alleviating the inflammatory damage. It is showed that MST1/2 inhibits YAP activity of the renal tubules to inhibit the chronic kidney disease (CKD) [30].

#### The role of MST1/2 in immune

*MST1/2 is involved in the regulation of innate immunity*. MST1/2 plays an important role in regulating the innate immune. It is reported that MST1/2 is involved in macrophage phagocytosis [31]. CaMKII-MST1/2-Rac signaling pathway is activated by toll-like receptor 4 (TLR4), which enhances the bactericidal activity of the macrophages to involve in the innate immunity [32]. Thus, the inhibition of MST1/2 results in reduced ability of the macrophages to phagocytize Escherichia coli and Lactobacillus monocytogenes [29]. Accordingly, MST1 may involve in innate immune by enhancing the bactericidal activity of the macrophages.

In addition, it is reported that MST1/2 is expressed in eosinophils and associates with the apoptosis of the eosinophils [33]. However, no research has shown whether MST1 is involved in the regulation of the eosinophils. Furthermore, though MST1/2 is not expressed in neutrophils, the absence of MST1 inhibits the neutrophils penetrating the perivascular basement membrane and extravasating into the inflammatory tissues [34]. Moreover, meanwhile, the function of MST1 is inhibited resulting that the loss of the mitochondrial membrane potential of the neutrophils increases subsequently the susceptibility to apoptosis increases [13]. Accordingly, MST1 may involve in innate immune by promoting the exudation and the survival of the neutrophils.

MST1/2 is a selective driver of the CD8a+ antigen-presenting dendritic cells (DCS) and maintains the bioenergetic activities and the mitochondrial dynamics of CD8a+ DCs [35]. In addition, MST1/2 is closely related to the expression of IL-6 [36] and IL-12 in CD8α+DCs [35]. Moreover, CD4(+) Foxp3(+) Regulatory T cells (Treg cells) dynamic mobile contact with antigen-pulsed DCs that bearing stably associates naïve T cells in depends on MST1 [37]. Meanwhile, MST1 deficiency decreases DCs in the marginal area of the spleen and impairs the antigen presentation function of the CD8α+DCs [38]. Accordingly, MST1/2 may be involved in innate immune by regulating the function of DCs.

*MST1/2 is involved in the regulation of adaptive immunity*. It is confirmed that MST1/2 is involved in the regulation of the adaptive immune [39]. MST1 deficiency has impaired the adaptive immunity of individuals, such as the insufficient vaccination responses, the decrease of T and B lymphocytes [40], which leads to combined immunodeficiency [41].

MST1/2 is involved in the regulation of T cells. MST1/2 is a key factor of T cells [42] and involved in the regulation of T cells adhesion, migration, homing, and survival [31] to maintain the homeostasis of T cells [2]. Over-expression of MST1 leads to the increase of the proliferation and the migration of T cells [43]. In addition, MST1/2 is recruited by the constitutive interaction between Rap1 and Rassf5B (known as Nore1B/RAPL) which is Rap1-GTP binding protein to control the adhesion and the migration of T cells [44]. Moreover, MST1 mediates the migration of the mature thymocytes from the thymus to the periphery [45] and is closely related to the selection and the antigen self-recognition of thymocytes [38,46]. In MST1 ^−/−^ mice, the selection and the migration of thymocytes are impaired, and the antigen recognition efficiency is down-regulated [38].

MST1 maintains the homeostasis of the peripheral naïve T cells [47] and promotes the recognition of the antigens by naïve T cells [48]. However, MST1 inhibits the activation and the proliferation of naïve T cells through Rassf5-MST1 complex [47]. In addition, MST1-Foxos signaling pathway maintains the peripheral homeostasis of naïve T cells [49].

**Figure 2.**
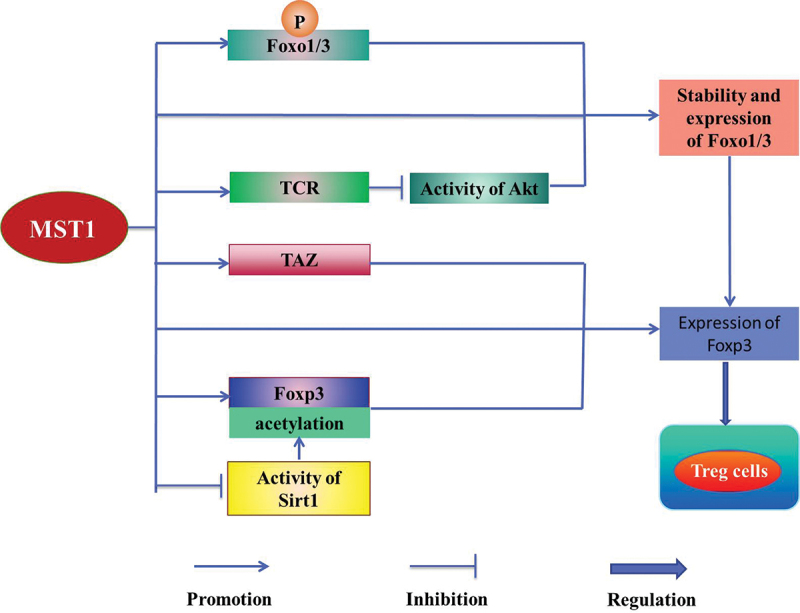
MST1/2 regulates Treg cells.

MST1/2 plays an important role in promoting the maturation and the function of Treg cells [31]. It is reported MST1/2 is involved in Treg cells [37] to maintain the balance between the activation and the tolerance of the immune [48], which affects the autoimmune inflammation and the normal immune function [14,29]. In addition, MST1 improves the differentiation and the function of Treg cells [14] by regulating the activities of Forkhead box o1/3 (Foxo1/3) [2], Sirt1 [50], TEAD1 [51], and Akt [14], meanwhile maintains the immune tolerance [29] by regulating the activation of STAT5 [52,53]. MST1 directly or indirectly enhances the stability of Foxo1/3 by phosphorylating Foxo1/3 or inhibiting activity of Akt mediated by antigen-specific T cell antibody (TCR) in the peripheral T cells, thereby increasing the expression of Foxp3 in the mice to regulate Treg cells [14]. In addition, MST1 increases the acetylation of Foxp3 directly or by inhibiting the activity of the human recombinant protein 1 (Sirt1) to increase the stability of Foxp3 [50], which improves the activity of Foxp3 to regulate Treg cells [29,52] (Figure 2).

Foxo1/3 Forkhead box o1/3; Treg cells Regulatory T cell; Foxp3 A special marker of Treg cells; Sirt1 Human recombinant; TCR Antigen-specific T cell antibody; Akt Protein kinase B; TAZ The homolog of Yorkie, also known as WWTR1

MST1 directly or indirectly enhances the stability of Foxo1/3 by phosphorylating Foxo1/3 or inhibiting activity of Akt mediated by TCR, which increases the expression of Foxp3. In addition, MST1 increases the acetylation of Foxp3 directly or by inhibiting the activity of Sirt1 to increase the activity of Foxp3 to regulate Treg cells. Meanwhile, MST1 directly or through MST1-TAZ axis promotes the expression of Foxp3 to regulate Treg cells.

Meanwhile, MST1/2 directly or through MST1/2-TAZ axis promotes the expression of Foxp3 to regulate the reciprocal differentiation of Treg cells and Th17 cells [29] (Figure 2). Foxp3+ Treg cells and Th17 cells, as a pair of balancers [29], play an important role in maintaining the homeostasis of cells [2]. It is reported that Foxp3+ Treg cells attenuate the proliferation of T cells and the secretion of pro-inflammatory cytokines, mediating the immune tolerance [29] and the immunosuppression [54]. In contrast, the pro-inflammatory role of the Th17 cells protects the hosts against the infection [2]. MST1/2-TAZ signaling inhibits the development of Th17 cells, but improves the differentiation of Treg cells [55]. Accordingly, MST1/2 plays a key role in maintaining the homeostasis of T cells [2,29].

The homeostasis and the function of T cells are frequently impaired with the absence of MST1 resulting that the hosts present susceptibility to the immunodeficiency syndrome, which causes various diseases because of recurrent bacterial and viral infections [13,41,56], such as lung infections, candidiasis, and non-degenerative skin warts [57]. Moreover, the deficiency of MST1 causes hypergammaglobulinemia with the production of autoantibody [42], which leads to the autoimmune diseases such as Sjogren’s syndrome and colitis [14]; meanwhile, the hypergammaglobulinemia is associated with increased levels of IgG, IgA, and IgE [41]. It is reported that the hypergammaglobulinemia is likely attributed to the inhibition of MST1-Foxo1 signaling pathway which decreases the expression of Foxp3 to impair the function of the Treg cells, ultimately leads to the inability to maintain the dominant tolerance [41]. Accordingly, MST1 is an important regulator of Treg cells and is critical for preventing the autoimmune diseases and maintaining the immune homeostasis [55]. The deficiency of MST1 causes the immunodeficiency syndrome [14] or the autoimmune diseases. Accordingly, MST1/2 may be involved in the adaptive immune by regulating the function of T cells.

**Figure 3.**
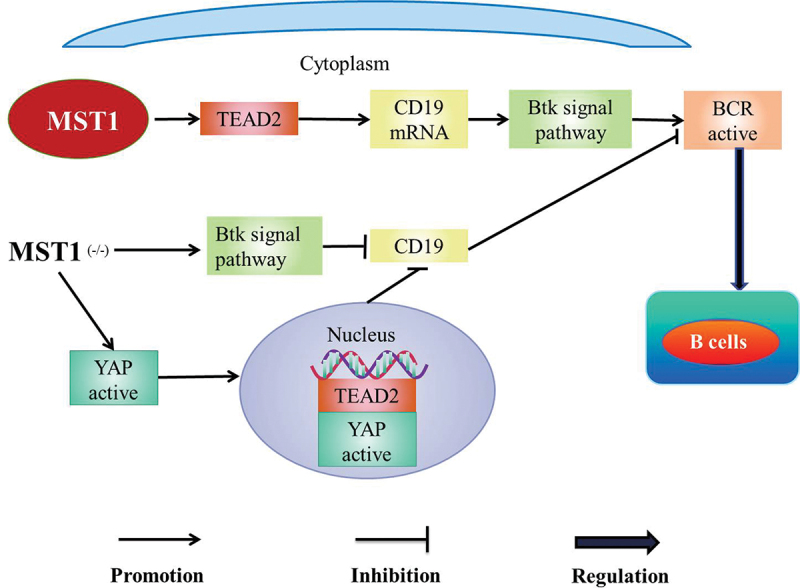
MST1-Btk pathway.

MST1/2 is involved in the regulation of B cells. MST1 promotes the diffusion and the transportation of B cells [58]. In addition, MST1 co-promotes the development and the activation of B cells with B cell antigen receptor (BCR) [59]. MST1 positively regulates BCR signal by activating Bruton tyrosine kinase (Btk) signal pathway, which leads to the decrease of B cells in the marginal zone (MZ) and the increase of B cells in the germinal center (GC) [60] (Figure 3). Meanwhile, mouse MST1/Wiskott-Aldrich syndrome protein (WASP) is knocked out (DKO), early activation events of B cells such as BCR signaling, BCR clustering, and B-cells spreading are severely impaired and the development of B cells is seriously hindered in bone marrow [59]. MST1/2 promotes the maturation of B cells in the white pulp of the spleen and then B cells gain the ability of recycling to the lymph nodes or the bone marrow [58]. Meanwhile, MST1/2 promotes the effective transportation of follicular B cells to the splenic red pulp to produce marginal B cells [58]. The number of B cells decreases [38] and the development of B cells defects in mice with MST1 ^(-/-)^ [61] besides the germinal center is dysregulated in the spleen margin area, which eventually leads to primary immunodeficiency [62]. Accordingly, MST1/2 may be involved in the adaptive immune by regulating B cells.

BCR B cell antigen receptor; TEAD1 TEA domain family member 1; Btk Bruton tyrosine kinase

MST1 up-regulates the messenger RNA level of CD19 by regulating TEAD2, which mediates Btk pathway activating BCR signaling to regulate B cells. While MST1 is deficient, the expression of CD19 is decreased through Btk pathway. In addition, the deficiency of MST1 leads to the increase of YAP expression and the collection of YAP in the nucleus. Moreover, YAP binds and activates TEAD2 to inhibit the expression of CD19, which inhibits the activity of BCR signal and weakens the function of MST1 to regulate B cells.

MST1/2 is involved in regulation of lymphocytes. MST1/2 regulates the survival [48], polarity, adhesion [63], and transportation [58] of lymphocytes through the non-canonical Hippo pathway [48]. It is confirmed that MST1 is the most abundant in the lymphoid organs including the thymus, the spleen, and the lymph nodes [2]. MST1 promotes the transportation of lymphocytes to the secondary lymphoid organs [58]. Meanwhile, MST1 is lacking in the mouse lymphocytes, the spleen may become swollen [14] and the peripheral lymphoid tissue atrophy [38]. In addition, MST1 is a key effector of Rassf5 which mediates effective transport of the immune cells [63]. The levels of Rassf5 decreased significantly [47] and lymphocytes present profound transport defect in MST1 ^(-/-)^ lymphocytes [64]. Moreover, MST1 promotes the adhesion of lymphocytes by transmitting Rap1-RAPL signals [63]. It is reported MST1 ^(-/-)^ lymphocytes are incapable to adhere firmly to the high endothelial venules through alpha4 integrin, which results the significant reduction of time that lymphocytes stop on the endothelial cells and significantly decrease of the homing ability of lymphocytes [38]. Meanwhile, MST1/2 promotes the survival of lymphocytes [63]. Dysfunction of MST1 leads to the decrease of the mitochondrial membrane potential and the increase of susceptibility to apoptosis of lymphocytes [13]. Besides, MST1/2^(-/-)^ leads to severe lymphopenia [38,65]. Accordingly, MST1/2 may be involved in the immune by regulating the function of lymphocytes.

## MST1/2 and signaling pathway

Hippo signaling pathway is evolutionarily conserved [66], which is involved in the regulation of proliferation and apoptosis of cells [66,67]. The major role of Hippo signaling pathway may be regulating the immunity in the mammals [68], which is essential for maintaining the homeostasis of the immune system [29]. MST1/2 is the core kinase of Hippo signaling pathway and cross-talks with the various signaling pathways [29] such as MST1-JNK signaling pathway [22], MST1-NF-κB signaling pathway [69], and MST1/2-Rac signaling pathway [32] in the various immune cells [29]. In addition, MST1/2 is involved in the regulation of the immunity [70] and the inflammation in Hippo pathway [12].

**Figure 4.**
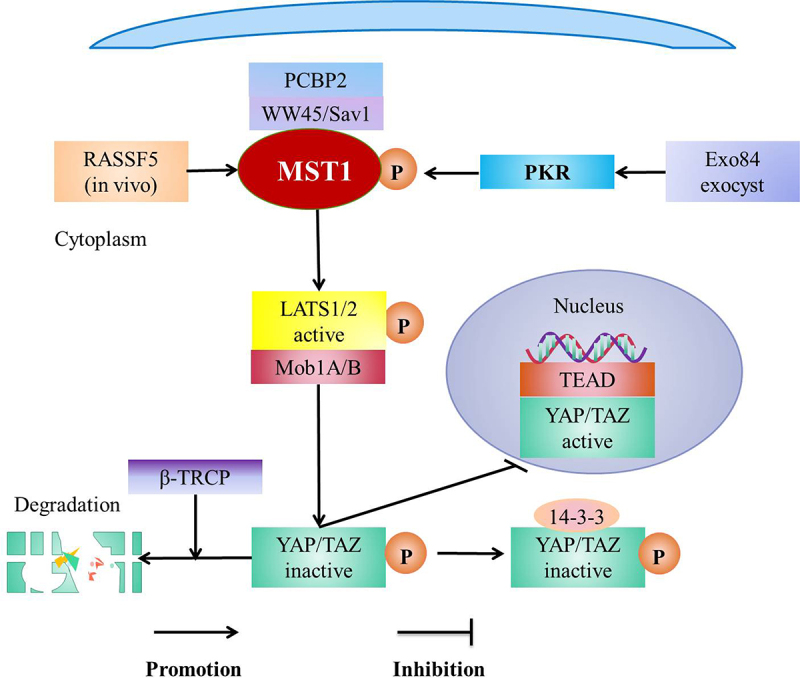
Hippo pathway and regulation of Hippo pathway.

Hippo pathway is composed of a kinase cascade [66], including MST1/2 and its scaffold protein WW domain protein 1 (WW45)/Salvador1 (SAV1), large tumor suppressor kinases 1 and 2 (LATS1/2, Warts homologs) and its scaffold-protein MPs 1 binding compounds l A and B (Mob1A/B), and downstream effector YAP/TAZ [19,30]. The core components of Hippo pathway are MST1/2, LATS1/2 and their respective adaptor proteins [19]. In addition, the main effect factors of Hippo pathway are YAP and TAZ [30]. The MST1/2-WW45/SAV1 complex phosphorylates and activates LATS1/2-Mob1A/B complex which then phosphorylates YAP/TAZ and then the phosphorylated YAP/TAZ is degraded by β-TRCP or isolated in the cytoplasm by 14-3-3 [29]. MST1/2 activates Hippo signaling pathway [71] to decrease the accumulation of nuclear YAP and inhibit the retention of TAZ in the mesothelial cells [72]. Meanwhile, MST1/2 is inactivated to close Hippo pathway, which leads to the accumulation of YAP/TAZ in the nucleus and then YAP/TAZ binds to TEAD family transcription factors [73], subsequently initiates the expression of downstream genes [29,66] (Figure 4). In the mammals, MST1/2 regulates LATS1/2 and NDR1/2 by scaffolding WW45/Sav1 [19]. In addition, NDR1/2 is the same kinase family as LATS [45] and is a branch of LATS1/2 [2].

WW45 Scaffold protein WW domain protein 1 of MST1/2; SAV1 Salvador 1, a homolog of SAV; PCBP2 Poly(rC) binding protein 2; RASSF5 (NORE1A/RAPL) Rap1-GTP binding protein, it is the RAS-association (RA) domain family, contains the SARAH domain and is a key regulator of MST1/2; PKR Immune kinase protein kinase R; LATS1/2 Large tumor suppressor kinases 1 and 2, Warts homologs; Mob1A/B Scaffold-protein MPs 1 binding compounds l A and B of LATS1/2; TEAD TEA domain family member; Exocyst An evolutionarily conserved protein complex that regulates vesicular trafficking and scaffolds signal transduction

The MST1/2-WW45/SAV1 complex phosphorylates and activates LATS1/2-Mob1A/B complex which then phosphorylates YAP/TAZ and then the phosphorylated YAP/TAZ is degraded by β-TRCP or isolated in the cytoplasm by 14-3-3. MST1/2 activates Hippo pathway to decrease the accumulation of nuclear YAP and inhibits the retention of TAZ in the mesothelial cells. However, inactivation of MST1 leads to the accumulation of YAP/TAZ in the nucleus and then YAP/TAZ binds to TEAD family transcription factors, subsequently initiates the expression of downstream gene. Rassf5 activates MST1/2 in the vivo. PCBP2 specifically interacts with SAV1 to activate MST1. PKR phosphorylates and activates MST1 by Exo84 exocyst.

### The signaling pathways of MST1/2 related to inflammation

#### MST1-JNK signaling pathway

The activation of MST1-JNK signaling pathway aggravates the inflammation in the myocardial cells [21], liver cells [74], and the neurons [22]. It is reported that the activity of MST1 is up-regulated after LPS treatment [75] to promote the activation of JNK pathway [21], leading to the up-regulation of SRV2 [22], which subsequently increases the levels of fission-promoting factor mitochondrial Kinetic associated protein (Drp1) [21]. Furthermore, the phosphorylated JNK is transported to the nucleus to increase the expression of the drp1 receptor (Mff) [22,76]. Drp1 migrates from the cytoplasm to the mitochondrial surface and binds with Mff on the mitochondrial outer membrane to recruit Drp1 [21]. Drp1 subsequently forms a GPT-dependent contraction ring which divides the mitochondria into several fragments to promote the mitochondrial fission, the mitochondrial potential collapsing, and the mitochondrial permeability transition pore (mPTPs) opening [21]. The mitochondrial pro-apoptotic mediators (such as cyt-c [77] and Smac) leak into the cytoplasm after mPTPs being opened [77] to active the apoptotic executive caspase-3, caspase-9 and rapidly down-regulate the activities of antioxidants such as SOD, GSH, and GPX [78], which leads to the mitochondrial apoptosis [76], thus the apoptosis of the myocardial cells and BV-2 cells occurring [21,22] (Figure 1).

Sirt3 is a key factor for the survival of the microglia. It is reported that Sirt3 inhibits the expression of SRV2, which maintains the antioxidant levels to inhibit the cleavage and the apoptosis of the mitochondria through MST1-JNK signaling pathway, which is effective in preventing microglia death induced by the neuroinflammation [22] (Figure 1).

**Figure 5.**
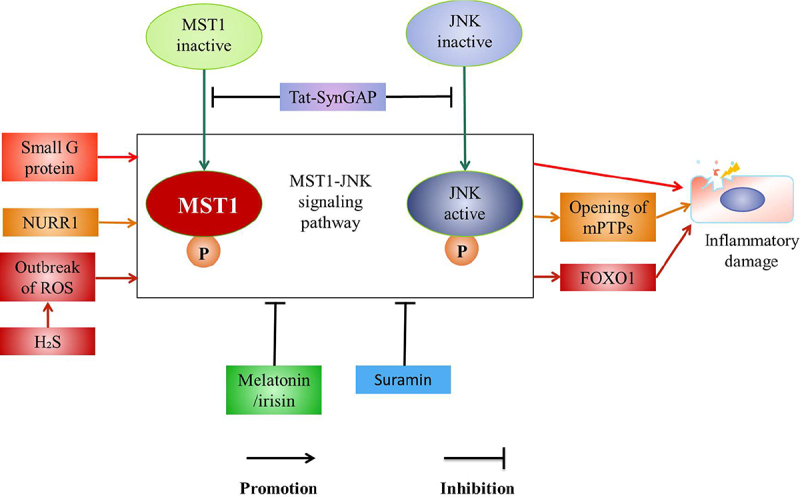
The regulation of MST1-JNK pathway.

MST1-JNK signaling pathway is regulated by the various factors, such as small G protein [79], nuclear receptor-associated protein 1 (NURR1) [80], and hydrogen sulfide (H_2_S) [81]. These factors activate MST1-JNK signaling pathway; meanwhile, melatonin/irisin co-treatment [75], Suramin [74], and Tat-SynGAP (670-685aa) inhibit the activation of this pathway [79]. It is suggested that small G protein is involved in the regulation of the tissue damage via promoting the activation of MST1-JNK signaling pathway [79]. In addition, NURR1 promotes the activation of MST1-JNK-mPTP pathway to decrease the mitochondrial membrane potential, which promotes the opening of mPTPs, and increases the oxidative stress, which results in the myocardial cell necrosis [80]. Exposure of H_2_S causes outbreak of reactive oxygen species (ROS) to activate JNK-MST1-Foxo1 pathway to aggravate the inflammatory damage [81]. Meanwhile, Tat-SynGAP inhibits MST1-JNK signaling pathway by inhibiting the cleavage of caspase-3 and the phosphorylation of MST1 or JNK, and promoting the expression of angiogenesis-related molecules VEGF, Ang-l to reduce the apoptosis of the neuronal and the volume of the cerebral infarction to maintain the stability of the vascular and the integrity of BBB [79]. Moreover, the Melatonin/irisin co-treatment significantly inhibits MST1-JNK pathway to prevent the death of the myocardial cells [75]. It is reported that the expression of MST1 aggravates LPS-mediated hepatocytes death [74], meanwhile the loss of MST1 weakens LPS-mediated mitochondrial damage [74,82]. In addition, Suramin reduces the expression of MST1 by inhibiting JNK-MST1 signaling pathway to inhibit the mitochondrial oxidative stress and block the caspase-9-mediated mitochondrial apoptosis to exert anti-apoptotic or anti-inflammatory effects and maintain the viability of the liver cells [74]. Accordingly, the activation of MST1-JNK pathway aggravates the inflammatory injury [22] and the inflammatory injury is significantly improved after this pathway being inhibited [75,79] (Figure 5).

JNK c-Jun N-terminal kinase; NURR1 Nuclear receptor-associated protein 1; mPTP Mitochondrial permeability transition pore; H_2_S Hydrogen sulfide; ROS Reactive oxygen species; FOXO Forkhead box o; Tat-SynGAP 670-685aa

Small G protein promotes the activation of MST1-JNK pathway to aggravate the inflammatory damage. In addition, NURR1 promotes the activation of MST1-JNK to decrease the mitochondrial membrane potential and promote the opening of mPTPs, thus resulting in inflammatory damage. Exposure of H2S causes outbreak of ROS to activate JNK-MST1-Foxo1 pathway, which aggravates the inflammatory damage. In addition, Tat-SynGAP inhibits the phosphorylation of MST1 or JNK to inhibit MST1-JNK pathway, which reduces the inflammatory damage. Moreover, the Melatonin/irisin co-treatment significantly inhibits MST1-JNK pathway to prevent the inflammatory damage. Furthermore, Suramin reduces the expression of MST1 by inhibiting JNK-MST1 signaling pathway to relieve inflammatory damage.

#### MST1-NF-κB signaling pathway

The nuclear factor-κB (NF-κB) pathway plays an important role in the inflammation [69]. MST1 presents a negative feedback regulation effect on NF-κB pathway induced by LPS [10]. It is reported that MST1 negatively regulates NF-κB signaling pathway mediated by TLR4 in the process of the macrophage activation to reduce the production of the pro-inflammatory cytokines such as IL-1β, IL-6, and TNF-α [2,10]. In addition, leonurine (LNR) inhibits NF-κB signaling pathway by up-regulating the expression of MST1 to inhibit LPS-induced trophoblastic inflammation response [83]. Paradoxically, MST1 promotes the inflammatory response and the mitochondrial dysfunction directly or by activating NF-κB signaling pathway in the process of aneurysm formation, finally to aggravate the inflammatory damage [84]. It seems that the effect of MST1 on NF-κB signaling pathway is bidirectional.

#### MST1-mROS signaling pathway

MST1-mROS signaling pathway is related to the inflammation and the death of the myocardial cells [18]. With I/R and hypoxia-reoxygenation (HR) injury, the transcription of Interleukin-2 (IL-2), IL-8, and TNF-α rapidly increase [18]. In addition, GSH, GPX, and SOD are rapidly downregulated [18]. These alterations are abolished by SRV2 deletion [18]. The levels of SRV2 in the myocardial cells increase after I/R and HR injury to active MST1-mROS signaling pathway through up-regulating MST1 and mROS, which aggravates the inflammation of the cardiomyocytes and promotes the death of the cardiomyocytes [18].

#### MST1-Foxo3 signaling pathway

It is suggested that LPS increases the expression of MST1 and p-Foxo3 protein to active MST1-Foxo3 signaling pathway, which causes the inflammatory reaction, leading to the apoptosis of hippocampal neurons [85]. Tanshinol (TSL) inhibits the expression of MST1 and p-Foxo3 to improve working memory by significantly inhibiting the levels of IL-1β, IL-6, and TNF-α in the plasma and hippocampus [85]. Accordingly, the activation of MST1-Foxo3 pathway aggravates the inflammatory injury and the inflammatory injury is significantly improved after this pathway being inhibited.

### Signaling pathways of MST1/2 related to immunity

#### MST1/2-rac signaling pathway

**Figure 6.**
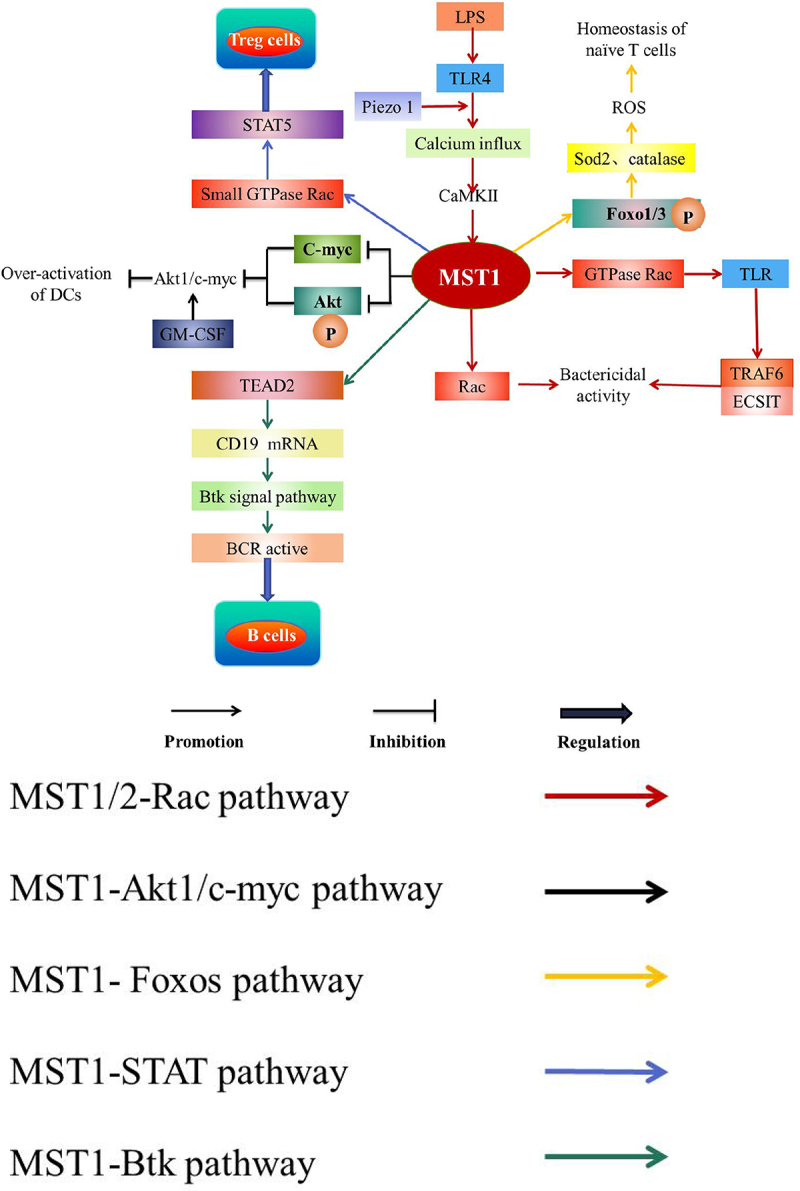
Pathways of MST1 related to immunity.

TLR4 augments the bactericidal activity of the macrophages through the mechanical sensor Piezo 1; meanwhile, LPS stimulates TLR4 to induce calcium influx mediated by Piezo 1 to active CaMKII-MST1/2-Rac pathway to ingest and kill the pathogens, thus driving the innate immune response of the microbial infection [32]. In addition, MST1/2 promotes TLR to trigger the assembly of TRAF6-ECSIT (pathway mediator) complex by activating GTPase Rac, leading to the recruitment of mitochondria to phagosomes and increase of mROS, which finally kills the intracellular bacteria [86] (Figure 6). Accordingly, MST1 is involved in TLR-MST1/2-Rac signal axis [86] and CaMKII-MST1/2-Rac axis [32] to augment the bactericidal activity.

LPS Lipopolysaccharide; TLR Toll-like receptor; Akt Protein kinase B, PKB; GM-CSF Granulocyte-monocyte colony-stimulating factor; Sod2 Superoxide dismutase 2; ROS Reactive oxygen species; TEAD2 TEA domain family member 2; Btk Bruton tyrosine kinase; BCR B cell antigen receptor

LPS stimulates TLR4 to induce calcium influx mediated by Piezo 1 to active CaMKII-MST1/2-Rac pathway, which ingests and kills the pathogens. In addition, MST1 promotes TLR to trigger the assembly of TRAF6-ECSIT (pathway mediator) complex by activating GTPase Rac, which finally kills the intracellular bacteria. Accordingly, MST1 augments the bactericidal activity through MST1-Rac pathway. In addition, MST1 inhibits the phosphorylation of Akt1 and c-myc protein levels to down-regulate response of Akt1/c-myc axis to GM-CSF to inhibit the over-activation of DCs. Moreover, MST1-Foxos pathway is essential for the survival of naïve T cells. Furthermore, MST1 phosphorylates and activates Foxo1/3, which up-regulates Sod2 and catalase to maintain the level of ROS, thereby maintaining the peripheral homeostasis of naïve T cells. MST1 in Treg cells activates the small GTPase Rac to subsequently active STAT5, which regulates Treg cells. MST1 up-regulates the messenger RNA level of CD19 by regulating TEAD2, which mediates Btk pathway activating BCR signaling to regulate B cells.

#### MST1-Akt1/c-myc pathway

MST1 inhibits over-activation of DCs [87]. It is reported that MST1 inhibits the phosphorylation of Akt1 and c-myc protein levels to down-regulate Akt1/c-myc axis response to granulocyte-monocyte colony-stimulating factor (GM-CSF) to inhibit the over-activation of DCs [87] (Figure 6). In addition, the absence of MST1 in DCs leads to increase the expression of the cell surface molecules such as B7 and MHC class II and the pro-inflammatory cytokines such as IL-23, TNF-α, and IL-12p40, indicating that MST1-deficiency may induce the hyperactivation of DCs [87].

#### MST1-Foxos signaling pathway

MST1-Foxos signaling pathway is essential for naïve T cells to survive [49]. MST1 phosphorylates and activates Foxo1/3 in the peripheral blood T cells of the mice, which up-regulates the targets of Foxos such as superoxide dismutase 2 (Sod2) and catalase to maintain the levels of ROS and protect naïve T cells from oxidative stress, thereby maintaining the peripheral homeostasis of naïve T cells [2,49] (Figure 6). Meanwhile, the inactivation of MST1 inhibits the phosphorylation of Foxos to inhibit the downstream signaling pathway of MST1-Foxos [88], which finally affects the peripheral homeostasis of naïve T cells [49].

#### MST1-STAT signaling pathway

MST1 is a signal-dependent amplifier of IL-2-STAT5 activity in Treg cells [53]. IL-2 signaling is considered a major regulator for controlling the homeostasis and function of Treg cells [89,90]. MST1/2 in Treg cells activates the small GTPase Rac to active STAT5 under the action of IL-2, which maintains the subpopulation of highly inhibited phosphorylated-STAT5+Treg cells and the pool of stable Treg cells [53] (Figure 6). Recently, studies discover a highly suppressive p-STAT5+ Treg cell subpopulation is crucial for the suppression of autoreactive T cells and incipient autoimmunity [91].

#### MST1-Btk signaling pathway

MST1 up-regulates the messenger RNA levels of CD19 by regulating TEAD2, which mediates Btk signaling pathway activating BCR signaling to regulate MZ and GC B cells [61] (Figure 6). With MST1 deficient, the expression of CD19 which maintains the normal function of B cells is decreased through Btk signaling pathway [61]. In addition, the deficiency of MST1 leads to the increase of YAP expression and the collection of YAP in the nucleus [61]. Moreover, YAP binds and activates TEAD2 to inhibit the expression of CD19, which inhibits the activity of BCR signal and weakens the function of MST1 to regulate B cells [61] (Figure 3).

## MST1/2 and upstream regulatory factors

MST1/2 is activated undergo homodimerization or inactivation undergo heterodimerization through SARAH domain contained in non-catalytic polypeptides Rassf1–6 or Sav1/WW45 [5].

There are a variety of factors that regulates the activity of MST1, such as Poly(RC) binding protein 2 (PCBP2) [71], Rassf5 (in vivo) [92], and immune kinase protein kinase R (PKR) [93]. PCBP2 specifically interacts with scaffold protein SAV1 to prevent proteolytic cleavage of MST1 [71]. PKR assembles and phosphorylates MST1 by Exo84 exocyst under the viral infection to activate MST1 [93]. In addition, Rassf is a key regulator of MST1/2 [94], it belongs to RAS-association (RA) domain family [95]. It is reported that Rassf1–6 binds MST1 and regulates the activity of MST1 [95]. Rassf5 activates MST1/2 in the vivo [92] (Figure 4). In vivo, membrane-anchored Ras dimer (or nanoclusters) promotes the heterodimerization of SARAH domain [96], meanwhile the homodimerized domain of MST1/2 is exposed and trans-autophosphorylated to be activated [96,97], which is consistent with the fact that the expression of MST1 in Treg cells decreases after Rassf5C being hypermethylated [42]. However, the membrane is lack and the homodimerization and the trans-autophosphorylation of MST1/2 domain is absent in vitro, thus Rassf5 inhibiting MST1/2 [97].

### MST1/2 regulatory factors related to inflammation

#### MST1 and TNF-α

The expression of MST1 increases rapidly under the influence of TNF-α in the vitro [98]. In the presence of TNF-α, knocking out MST1 weakens the mitochondrial dysfunction, reduces the cell oxidative stress, increases the cell vitality, and significantly inhibits TNF-α-induced inflammatory neuronal death [98]. However, in a TNF-α-induced inflammatory environment, the up-regulation of MST1 activates JNK pathway, leading to the mitochondrial homeostasis imbalance, the cell viability decrease, and the neuron death [98]. Accordingly, the simultaneous action of MST1 and TNF-α aggravates the inflammation, TNF-α cannot aggravate the injury with MST1 deficient [98].

#### MST1 and MIEF1

The inflammatory injury increases the levels of MST1 and the mitochondrial elongation factor 1 (MIEF1) to active MST1-MIEF1 pathway leading to the activation of ROS-related oxidative damage, the dysfunction of the mitochondria, and the initiation of the caspase-involved cell death [99]. Melatonin effectively inhibits MST1-MIEF1 axis and maintains the function of the mitochondrial to increase the survival rates of cells [99]. Accordingly, the activation of MST1-MEF1 axis damages the mitochondrial function and aggravates the inflammatory injury.

### MST1/2 regulatory factors related to immunity

#### MST1 and LFA-1

MST1 plays an important role in the recognition of lymphocyte function-related antigen 1 (LFA-1, also named to αLβ2 integrin) and its ligand intercellular adhesion molecule-1 (ICAM-1) in the medulla [100]. LFA-1 and ICAM-1 mediate the antigen-specific adhesion between T cells and antigen-presenting cells (APC) during the formation of the immune synapse (IS) [100]. The interaction between T cells and APC is impaired with MST1 is deficient [100]. In addition, MST1 regulates spatial distribution of LFA-1 and promotes the transmission of LFA-1 to the leading edge of lymphocytes [101] after chemokines stimulating T lymphocytes [63].

## Conclusion

MST1/2 plays an important role in the inflammation and the immunity. MST1/2 directly or indirectly aggravates the inflammatory injury and inhibits MST1/2 improve the prognosis of the inflammatory injury. MST1/2 may be considered a potential therapeutic target for the inflammatory injury of the body. However, MST1/2 may be a factor to alleviate the inflammatory damage in some cases. The reason for the difference of MST1/2 effect on the inflammation is still unclear. Meanwhile, MST1/2 is involved in regulating the innate and adaptive immunity and balancing the immune activation and tolerance to prevent the autoimmune inflammation and maintain the immune function. Patients with MST1/2 deficiency present susceptibility to the autoimmune diseases and the immunodeficiency syndrome.

MST1/2 is widely involved in the regulation of the inflammatory signaling pathways. The activation of MST1-JNK, MST1-mROS, and MST1-Foxo3 signaling pathways aggravates the inflammatory injury. MST1 negatively regulates NF-κB signaling pathway to reduce the inflammatory injury in the process of the macrophage activation. Paradoxically, MST1 activates NF-κB signaling pathway to aggravate the inflammatory injury in the process of aneurysm formation. The reason why MST1 shows two opposite effects on NF-κB signaling pathway is not yet clear.

MST1 maintains normal immune function via regulating immune-related signaling pathways to maintain the immune tolerance and prevent the autoimmune diseases. MST1 activates MST1/2-Rac axis to maintain effective bactericidal activity. In addition, MST1 inhibits over-activation of DCs through inhibiting Akt1/c-myc pathway. Moreover, MST1 activates Foxo1/3 pathway to maintain the peripheral homeostasis of naïve T cells and activates Bkt signal pathway to promote the development and activation of B cells.

Multiple factors are involved in the regulation of MST1/2 activity. PCBP2, Rassf5, and PKR activate MST1. In addition, TNF-α, MIEF1, and LPS promote MST1-mediated inflammation. Meanwhile, MST1 promotes antigen-specific adhesion by regulating LFA-1 and ICAM-1 in the immune response.

Taken together, MST1 is a therapeutic target for the inflammation and the immunity.

## Data Availability

All data are presented in this review.
